# Eating disorder NOS (EDNOS): an example of the troublesome “not otherwise specified” (NOS) category in DSM-IV

**DOI:** 10.1016/j.brat.2004.06.011

**Published:** 2005-06

**Authors:** Christopher G. Fairburn, Kristin Bohn

**Affiliations:** Department of Psychiatry, Warneford Hospital, Oxford University, Oxford OX3 7JX, UK

**Keywords:** DSM-IV, NOS, Eating disorder, Diagnosis, Classification, Clinical utility

## Abstract

The “Not Otherwise Specified” (NOS) category within DSM-IV is designed for disorders of clinical severity that are not specified within broad diagnostic classes. “NOS” diagnoses are intended to be residual categories and they tend to be neglected by researchers. This can be inappropriate. The problems associated with certain NOS diagnoses are well illustrated by “Eating Disorder NOS” (sometimes termed EDNOS), which is the most common category of eating disorder encountered in routine clinical practice yet it has barely been studied. Indeed, there has been no research on its treatment. Interim and longer-term conceptual and practical solutions to the anomalous status of eating disorder NOS are proposed including the creation of a new diagnosis termed “mixed eating disorder”. Several of these solutions are of relevance to NOS categories in general. All the solutions should fulfil criteria for clinical utility.

## 1 Introduction

The DSM-IV diagnosis “Eating Disorder Not Otherwise Specified” (eating disorder NOS) is much used by clinicians yet largely ignored by researchers. It is the most common category of eating disorder seen in outpatient settings yet there have been no studies of its treatment. Indeed, little has been written about eating disorder NOS. In this article we address this diagnosis from conceptual, clinical and empirical perspectives, our goals being to examine the diagnostic concept, highlight its clinical importance and suggest means of resolving its anomalous status.

## 2 “NOS” diagnoses in DSM and eating disorder NOS

Eating disorder NOS is an example of the “Not Otherwise Specified” (NOS) category in DSM-IV (American Psychiatric Association, 1994). Since the publication of DSM-III (American Psychiatric Association, 1980), the American Psychiatric Association's Diagnostic and Statistical Manual of Mental Disorders has included either “atypical” (in DSM-III) or “not otherwise specified” categories (in DSM-III-R (American Psychiatric Association, 1987) and DSM-IV), respectively in each broad diagnostic class in view of the difficulty covering every presentation encountered in clinical practice. These diagnoses are intended to “indicate a category within a class of disorders that is residual to the specific categories in that class…” (American Psychiatric Association, 1980, American Psychiatric Association, 1987).

Eating disorder NOS is the category in DSM-IV reserved for eating disorders of clinical severity that do not meet diagnostic criteria for either one of the two eating disorders recognised in DSM-IV, anorexia nervosa and bulimia nervosa. In common with other NOS diagnoses, it is a residual category. Thus, there are two steps in making a diagnosis of eating disorder NOS: first, it must be determined that there is an eating disorder of clinical severity; and then, it must be established that the diagnostic criteria of anorexia nervosa and bulimia nervosa are not met. This second step therefore involves diagnosis by exclusion: no positive diagnostic criteria for eating disorder NOS need to be fulfilled.

**Fig. 1:**
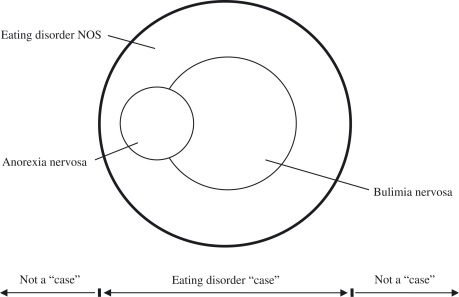
A schematic representation of the relationship between anorexia nervosa, bulimia nervosa and eating disorder NOS.

It is helpful to illustrate diagrammatically the relationship between the diagnoses anorexia nervosa, bulimia nervosa and eating disorder NOS (see Fig. 1). The two overlapping inner circles represent anorexia nervosa (the smaller circle) and bulimia nervosa (the larger circle) respectively, the area of potential overlap being that occupied by those people who would meet the diagnostic criteria for both disorders but for the DSM-IV “trumping” rule whereby the diagnosis of anorexia nervosa takes precedence over that of bulimia nervosa. Surrounding these two circles is an outer circle which defines the boundary of eating disorder “caseness”; that is, the boundary between having an eating disorder, a state of clinical significance, and having a lesser, non-clinical, problem with eating. It is this boundary that demarcates what is, and is not, an eating disorder. Within the outer circle, but outside the two inner circles, lies eating disorder NOS.

## 3 The characteristics of eating disorder NOS

### 3.1 Prevalence

Eating disorder NOS is the most common eating disorder diagnosis made in most outpatient settings other than those that attract highly specialist referrals. Table 1 shows the prevalence figures from four well-diagnosed adult samples. In each case eating disorder NOS was the most common diagnosis made, its weighted average prevalence being 60.0%. There have been two smaller-scale studies of adolescents with inconsistent findings (Bunnell, Shenker, Nussbaum, Jacobson, & Cooper, 1990; van der Ham, Meulman, van Strien, & van Engeland, 2004). It is important to note that the high proportion of eating disorder NOS cases in these samples is not due to laxity in defining what is a “case” for the data are from people seeking treatment in whom an eating disorder diagnosis has been substantiated by a clinician. Furthermore, as described below, there is now evidence that the severity of psychopathology and degree of secondary psychosocial impairment in those with eating disorder NOS are comparable to those seen in patients with anorexia nervosa or bulimia nervosa (Fairburn, Palmer et al., in preparation; Ricca et al., 2001; Turner & Bryant-Waugh, 2004).

**Table 1:** Prevalence of eating disorder NOS in samples of adult outpatients with eating disorders

	Sample size	DSM-IV diagnosis			Comments
		Anorexia nervosa %	Bulimia nervosa %	Eating disorder NOS %	
Martin et al. (2000)	175	19.4	22.9	57.7	BED 9.7% of full sample
Ricca et al. (2001)	189	24.9	24.9	50.3	BED 8.5% of full sample
Turner and Bryant-Waugh (2004)	190	5.8	23.7	70.5	EDE-based diagnoses. Sample excluded patients with BED
Fairburn, Palmer et al. (in preparation)	121	5.0	33.1	62.0	EDE-based diagnoses. Sample restricted to patients with a body mass index between 16.0 and 40.0. BED 5.8% of full sample
Weighted average		14.5	25.5	60.0	

BED—Binge eating disorder. EDE—Eating Disorder Examination (Fairburn and Cooper, 1993).

The prevalence of eating disorder NOS in the community is not clear. In large part this is because there are no positive diagnostic criteria for the diagnosis and so there is no agreed way of determining what constitutes a “case”. Instead, the figures quoted tend to be for the prevalence of participants with features suggestive of an eating disorder (other than anorexia nervosa or bulimia nervosa), no check being made that these features result in clinically significant distress or impairment (for example, Garfinkel et al., 1995b; Hay, Fairburn, & Doll, 1996), an essential requirement for a psychiatric diagnosis to be made (American Psychiatric Association, 1994).

### 3.2 Clinical features

Clinical descriptions of eating disorder NOS are consistent in stressing that most cases have clinical features that closely resemble those seen in anorexia nervosa and bulimia nervosa albeit at slightly different levels or in different combinations (Crow, Agras, Halmi, Mitchell, & Kraemer, 2002; Waller, 1993; Walsh & Garner, 1997). They also indicate that the majority of cases are young women, just as in anorexia nervosa and bulimia nervosa.

It is helpful to distinguish two subgroups within eating disorder NOS, although there is no sharp boundary between them (Fairburn & Walsh, 2002; Mitchell, Pyle, Hatsukami, & Eckert, 1986). In the first are cases that closely resemble anorexia nervosa or bulimia nervosa but just fail to meet their diagnostic thresholds; for example, their weight may be marginally above the limit for anorexia nervosa or their frequency of binge eating may be just too low for a diagnosis of bulimia nervosa. These cases may be viewed as “subthreshold” instances of anorexia nervosa or bulimia nervosa respectively. In the second group are cases in which the clinical features of anorexia nervosa and bulimia nervosa are combined in a different way to that seen in the two recognised syndromes. These cases are best described as “mixed”. Other terms have been used to describe such subgroups within eating disorder NOS including “subclinical” for the former subgroup, a term that is inappropriate given that these states are of clinical severity by definition; and “atypical” or “partial” for the second subgroup. Both the latter terms are problematic; the first because these states are not unusual and the second because of the implication that they are less severe than the full syndromes.

A recent development of relevance is the proposal that a third specific eating disorder be recognised in addition to anorexia nervosa and bulimia nervosa, effectively removing eligible cases from eating disorder NOS. This new diagnostic concept is termed “binge eating disorder” (BED) and is intended for people who experience recurrent episodes of binge eating in the absence of the extreme methods of weight control seen in bulimia nervosa and anorexia nervosa (American Psychiatric Association, 1994). This proposal was controversial when it was first suggested (Fairburn, Welch, & Hay, 1993; Spitzer et al., 1993) and divergent views on its merits still persist (Stunkard & Allison, 2003; Wilfley, Wilson, & Agras, 2003). As matters stand BED is not an established DSM-IV diagnosis and therefore eating disorders of this type remain under the rubric of eating disorder NOS. The data presented in Table 1 suggest that less than ten percent of adult eating disorder cases meet diagnostic criteria for BED.

There have been few systematic attempts to characterise the clinical features of patients with eating disorder NOS and compare them with those seen in anorexia nervosa and bulimia nervosa. Notable exceptions are three recent studies that have used the “gold standard” Eating Disorder Examination (EDE; Fairburn & Cooper, 1993) for this purpose. All three have confirmed that the characteristic clinical features of anorexia nervosa and bulimia nervosa are present and to a similar degree (Fairburn, Palmer, et al., in preparation; Ricca et al., 2001; Turner & Bryant-Waugh, 2004). Thus it has been found that patients with eating disorder NOS have the same distinctive behaviour and attitudes as patients with anorexia nervosa and bulimia nervosa, even to the extent that most individual EDE item ratings are remarkably similar (Turner & Bryant-Waugh, 2004). Our data show that this similarity extends to the duration of the eating disorder, severity of associated general psychiatric features and degree of secondary psychosocial impairment, especially when bulimia nervosa and eating disorder NOS are compared (Fairburn, Palmer et al., in preparation).

### 3.3 Course and response to treatment

Although there have been many studies of the course and outcome of anorexia nervosa and bulimia nervosa, few have considered eating disorder NOS as a specific outcome let alone have made clinical eating disorder NOS diagnoses. An exception is a recent study of the course of all forms of eating disorder which found that, although most participants retained an eating disorder (of some type), there was considerable cross-diagnostic flux with patients moving from one eating disorder diagnosis to another (Milos, Spindler, Schnyder, & Fairburn, submitted for publication). There has been just one study of the course of an unselected eating disorder NOS sample. It found that there was a “varied and persistent” course over 30 months and a low rate of recovery (Herzog, Hopkins, & Burns, 1993). As regards response to treatment, nothing is known for there have been no studies of the treatment of these patients (other than those of the small subgroup with BED).

## 4 Problems of nosology and neglect

This review of the prevalence, clinical features and course of eating disorder NOS highlights two inter-related problems. The first is the nosological status of eating disorder NOS. Clearly there is something amiss with the scheme for classifying eating disorders if the most common category is the “residual” one. The second problem is that the diagnosis is neglected despite being so common. The most striking example of this neglect is the fact that there have been no studies of its treatment.

It is possible that these two problems are related since the neglect of eating disorder NOS may be in part a consequence of its “NOS” status. “NOS” diagnoses in general are not much studied (Pincus, Wakefield Davis, & McQueen, 1999) and we have the impression that grant-giving bodies do not view them as a priority. They appear to be Cinderella states. In some countries this has a direct impact on patient care for the marginal status of NOS diagnoses even extends to restrictions on treatment provision or, at least, reimbursement for treatment (Herzog et al., 1993; Martin, Williamson, & Thaw, 2000). This could perhaps be justified were NOS states uncommon or mild but neither could be said to be true of eating disorder NOS.

## 5 Three potential solutions

Below we propose three potential solutions to these problems of nosology and neglect.

### 5.1 Relax the diagnostic criteria for anorexia nervosa and bulimia nervosa

The first solution is based on the premise that the high prevalence of eating disorder NOS cases is due to the DSM-IV diagnostic criteria for anorexia nervosa and bulimia nervosa being inappropriately strict. If true, some cases within eating disorder NOS would be better designated as cases of anorexia nervosa or bulimia nervosa. With reference to Fig. 1, this solution would involve expanding somewhat the two inner circles.

Done mindfully, relaxing the diagnostic criteria for anorexia nervosa and bulimia nervosa has much to commend it. Many clinicians and researchers have suggested that the DSM-IV criteria need to be adjusted in various ways (Crow et al., 2002; Garfinkel, Kennedy, & Kaplan, 1995a; Martin et al., 2000; Ramacciotti et al., 2002; Thaw, Williamson, & Martin, 2001) and in every instance this would have the effect of relaxing the current diagnostic thresholds. Such adjustments seem worth contemplating so long as the two diagnostic concepts are not materially altered. Two main suggestions have been made with respect to anorexia nervosa; the first being that the amenorrhoea criterion be dropped (Cachelin & Maher, 1998; Garfinkel et al., 1996; Watson & Andersen, 2003), and the second being that the “core psychopathology” be redefined to include states in which there is over-evaluation of controlling eating per se without requiring that there also be accompanying concerns about shape and weight (Palmer, 2003; Rieger, Touyz, Swain, & Beumont, 2001). Adjusting upward the weight threshold for anorexia nervosa is another option (Garfinkel et al., 1995a; Watson & Andersen, 2003), although only a marginal change could be accommodated without undermining the fundamental requirement that people with anorexia nervosa should be significantly underweight. With regard to bulimia nervosa the main bone of contention concerns the present twice-weekly threshold for the frequency of binge eating: it has been repeatedly argued that a lower minimum frequency would be more appropriate (Garfinkel et al., 1995b; Herzog, Norman, Rigotti, & Pepose, 1986; Wilson & Eldredge, 1991).

Changes of this type represent a fine-tuning of the existing diagnostic criteria rather than any radical change. They involve adding to the two established diagnostic concepts the “subthreshold” cases that exist within eating disorder NOS. Systematically applying all the above changes to our representative dataset (Fairburn, Palmer, et al., in preparation) indicates that their impact on the clinical prevalence of eating disorder NOS would be modest. This confirms our clinical impression that most cases of eating disorder NOS are of the “mixed” variety rather than “subthreshold”. Thaw and colleagues (2001) came to a similar conclusion, albeit using a convenience sample of eating disorder NOS cases.

### 5.2 Reclassify eating disorder NOS

The second solution is a response to the main shortcoming of the first; namely that it fails to address the fact that many cases within eating disorder NOS are mixed in nature. This solution is an elaboration and extension of the first. Subthreshold cases of anorexia nervosa and bulimia nervosa would be incorporated within these two diagnoses, respectively, as in the first solution, but in addition the remaining cases of eating disorder NOS would be reclassified as belonging to a new category of eating disorder. The majority of these cases would be mixed in character although a minority would fulfil diagnostic criteria for BED and might be best separated off. Thus, in summary, this solution would involve expanding anorexia nervosa and bulimia nervosa to embrace the subthreshold cases within eating disorder NOS and reallocating the remaining cases to a new diagnostic category, perhaps termed “mixed eating disorder”, or to BED.

### 5.3 The “transdiagnostic” solution

The third solution is the most radical. It would bring eating disorder NOS into the limelight by creating a single unitary diagnostic category “eating disorder” embracing anorexia nervosa, bulimia nervosa and eating disorder NOS without any subdivisions. The main argument for proposing a “transdiagnostic” solution of this type is that the current emphasis on subdividing the eating disorders (into anorexia nervosa and bulimia nervosa, each with their two subtypes, eating disorder NOS and possibly BED) detracts attention from the most striking characteristic of the eating disorders; namely, that far more unites the various forms of eating disorder than separates them (Fairburn & Harrison, 2003; Waller, 1993; Walsh & Garner, 1997). Thus, rather than focusing on differences between the eating disorders, there is a case for highlighting the many features that are shared by them and are largely peculiar to them. These include extreme dietary restraint and restriction, binge eating, self-induced vomiting and the misuse of laxatives, driven exercising, body checking and avoidance, and the over-evaluation of control over eating, shape and weight. These cross-diagnostic similarities become even more obvious if a longitudinal perspective is taken since, as noted above, patients do not adhere to their DSM-IV diagnosis over time; rather, they move between them (Fairburn and Harrison, 2003; Herzog et al., 1993; Milos et al., submitted for publication).

## 6 The need for positive diagnostic criteria

A second prerequisite for furthering research on the problems of patients with eating disorder NOS is the development of positive diagnostic criteria to delineate them. At present no specific features have to be present to make the diagnosis: rather, the sole requirement is that the person has an eating disorder of clinical severity other than anorexia nervosa or bulimia nervosa. In the absence of an agreed definition of what constitutes an “eating disorder,” this leaves considerable room for individual variation in diagnostic practice. This situation is quite different to that existing for anorexia nervosa and bulimia nervosa where a specific combination of clinical features must be present for either diagnosis to be made.

Given the existing diagnostic scheme, the challenge involved in formulating diagnostic criteria for eating disorder NOS lies in defining its outer “edges” (as illustrated in Fig. 1) since the inner boundaries, those of anorexia nervosa and bulimia nervosa, are already defined (although they could be adjusted as discussed above). Defining the outer edges of eating disorder NOS is possible. In principle, it requires identifying the type and level of eating disorder psychopathology that is typically associated with a clinically significant degree of secondary distress or disability. Good measures of eating disorder psychopathology are available (for example, the EDE) and we have developed a complementary measure of secondary clinical impairment that addresses the main domains of functioning affected by eating disorders; namely, mood, cognition, relationships, work and physical health. Used with appropriate samples, these instruments should in time provide the type of information needed to specify a threshold for the outer edges of eating disorder NOS, generating in the process an operational definition of what constitutes an “eating disorder”.

## 7 Discussion and broader implications

This paper has addressed the neglected DSM-IV diagnosis eating disorder NOS. Two misconceptions appear to keep eating disorder NOS on the margins of eating disorders. The first is the assumption that cases of eating disorder NOS are mild and therefore unimportant. The findings reviewed above indicate that this view is mistaken. The second misconception is that eating disorder NOS is uncommon. Data from eating disorder clinics give the lie to this view (see Table 1), but it is perhaps perpetuated by the “residual” status of NOS diagnoses in general.

We have suggested that two challenges have to be met for the problems of people with eating disorder NOS to get the attention that they deserve. One is that positive diagnostic criteria are needed and we have described a research strategy whereby they could be developed. It has not escaped our attention that doing this would be of value beyond simply defining eating disorder NOS and, in the process, what is an “eating disorder”. For example, it would provide a definition of caseness for epidemiological and clinical purposes and it would provide a new and clinically meaningful way of defining outcome for studies of treatment and natural course. At present most such studies ignore eating disorder NOS as a potential outcome thereby possibly inflating recovery rates. Having what constitutes an eating disorder delineated, with a good outcome being defined as being “over the edge” (i.e., no longer having an eating disorder), would provide a unified and consistent index of remission and recovery that would be the same whatever the eating disorder being studied. It might therefore make redundant the varied and somewhat inconsistent ways of representing outcome that are in use today. We are also aware that the proposed research strategy has broader implications too for it could be used to define the outer boundaries of other classes of psychiatric disorder.

The second challenge involves re-conceptualising the clinical problems that are currently categorised as eating disorder NOS. This is essential if the nosological anomaly of eating disorder NOS is to be resolved. Three solutions have been proposed. In the short term we favour the second solution because the first ignores the fact that many of the cases within eating disorder NOS are of the mixed variety. It involves relaxing the diagnostic criteria for anorexia nervosa and bulimia nervosa to extract the subthreshold cases from eating disorder NOS, the remaining cases being re-classified as cases of mixed eating disorder or BED. We are aware that the introduction of a new eating disorder diagnosis is inconsistent with the conservative spirit of DSM-IV but, as Nielsen and Palmer point out, “There is room for a measure of conservatism but we cannot be satisfied until the EDNOS issue is more adequately addressed” (Nielsen & Palmer, 2003, p. 162).

The second solution would have the effect of eliminating the concept of eating disorder NOS, at least for the meantime. The diagnosis would re-appear, however, once specific criteria for the “edges” were formulated (i.e., criteria for what constitutes an eating disorder) since in practice some “cases” of clinical severity would inevitably be encountered that would fall outside the new boundary, however well it was defined. These cases should be modest in number, rendering eating disorder NOS a small residual category, as NOS categories are intended to be.

We acknowledge that this re-classification of the cases within eating disorder NOS is something of a sleight of hand, but it is a sleight of hand with a purpose since it is intended to place these cases in specific and appropriate diagnostic categories. This might enhance the credibility and usefulness of the scheme for classifying eating disorders and, hopefully, it might also facilitate research on these problems including research on their treatment. We believe that this proposal would fulfil the First et al. (2004) criteria for “clinical utility”.

As regards the “transdiagnostic” solution, we believe that in the longer term it has the most to recommend it. The existing scheme for classifying eating disorders is a historical accident that is a poor reflection of clinical reality. The transdiagnostic solution would encourage and permit the classification of eating disorders to be examined afresh. The collection of good transdiagnostic data, particularly cross-diagnostic information on course and response to treatment, is needed if new clinically informative subdivisions are to be identified.
